# Meat-Inspection1Read before the American Veterinary Medical Association at New York, in September, 1899, introducing the subject of “Meat-inspection.”

**Published:** 1899-12

**Authors:** E. B. Ackerman

**Affiliations:** Brooklyn, N. Y.


### MEAT-INSPECTION.[1]

By E. B. Ackerman, D.V.S.,
BROOKLYN, N. Y.

As a representative of our local Department of Health I have been asked by our worthy Secretary to open the discussion upon "Meat-inspection," as started at the meeting held at Omaha, Neb., in September, 1898.

I regret to say that I was unable to be present at that meeting to take part in person in the discussion of this subject, but thanks to our Publication Committee a lengthy and full report has been published upon the various topics of this subject as taken up by various members of this Association.

[1] Read before the American Veterinary Medical Association at New York, in September, 1899, introducing the subject of "Meat-inspection."

Possibly the best means of opening the discussion this year would be a synopsis or review of the papers of last year, principally by title; taking them up in the order read they would include Dr. Hoskins' paper on "Methods of Cultivating Public Opinion as to the Necessity of Municipal Meat-inspection." Certainly, this was an excellent start on the subject, because it is quite necessary to get a theme before the public generally if you want it acted upon with any degree of interest, and I think Philadelphia veterinarians are to be congratulated upon the way they have put the subject before the people of that city, in the interest that has been awakened there. It is to be regretted that Dr. Hoskins, who had the honor of being the nominee for Mayor of the city of Philadelphia on the Democratic ticket last year, could not have been elected. Had he succeeded, I am sure we would have seen a revolution in the system of meat-inspection and milk-inspection that would have been a credit to his city and a worthy model for other cities to copy. It would also have been an honor to the veterinary profession that we might well be proud of.

But once get the subject before the people and press, and agitate it in the local health boards; then Dr. Pearson's remarks on the "Necessity of Consolidation of Slaughter-houses into large Abattoirs under Municipal Control, and Disposition of the Flesh of Tuberculous Cattle" would apply; and that is, from a practical point of view, the most important part, as expressed by Dr. Pearson in his able argument. The only way to make possible the systematic and thorough inspection of meat is through public abattoirs.

Much time and thought were given by me upon the subject of meat-inspection when I had charge of that department in the Board of Health in the old City of Brooklyn. In 1894 I addressed the following communication in my annual report to the Commissioner of Health upon the sanitary side of meat-inspection, public abattoirs, and disposition of refuse or offal: "I approve of public abattoirs located on the water-front (at Wallabout Market). There would be the opportunity of delivering cattle and animals for slaughter direct from the boat to the slaughter-house; in this way the danger of over-heating cattle by driving them through the streets in hot weather would be avoided, and there would be no opportunity for them to escape and rush madly through the streets, as occasionally is the case now. . . . The public abattoirs should be built with slaughtering-stands and with asphalt floors, so that each firm could hire as many stands provided with ice-boxes or ice-box space as they needed. At the proposed market the offal could all be handled by improved machinery, the fat treated, while fresh and inodorous, in new and improved rendering-tanks, and the offal and blood digested, ground, and dried as fast as made, making a good brand of fertilizer. All this could be done without odor, and could be made a large source of revenue to the city. To sum up, we would then have a public market, centrally located, equalled by none in the country; an abattoir clean and sanitary, easily watched and regulated. As a source of revenue to the city it would have the wharfage, rent from slaughtering-stalls, ice-boxes, and offices, an income from the manufacturer of fertilizer and fat-rendering establishments, together with the saving of salaries of several inspectors, with a much better system of inspection than now exists, beside a saving from carting, towing, and dumping of offal, and the abatement of the nuisance arising from the removal of said offal.''

As slaughtering exists to-day in our scattered system of slaughter-houses, it would require an army of inspectors to be on hand at all hours of the day and night, as well as Sundays, to see all the cattle; and as it is now, there are certain cows that are held and run in after the inspectors leave, if they are present at all. Thus the resolutions offered at our annual meeting in 1897 met with my hearty approval.

Dr. Charles A. Cary's "Reasons for Meat-inspection" added largely to our knowledge, and his extracts from the law on the municipal authority to compel butchers to slaughter in public abattoirs is very valuable, and goes a long way toward the aid in starting this and other municipalities.

"Public Market Inspection," by Dr. Heintzman, of New Orleans, giving us the regulations of his city, was also a very valuable addition to the list of papers.

To sum up, then, I could say the inspection of meat in municipalities should be divided into three classes of inspection, viz., first, slaughter-house inspection; second, inspection of wholesale and commission houses; and, third, inspection of retail butcher-shops and markets. Taking the last first, it would consist of an inspection that would have to do with the sanitary condition of the store and ice-boxes and the condition of the meat found therein, both fresh, salt, and corned. In this inspection it might be well for the inspectors to use such a formula as suggested by Dr. Heintzman, of alcohol, hydrochloric acid, and ether in all doubtful cases. In the inspection in Brooklyn, when I had it in charge, I divided the city into districts, principally by wards, and had the inspectors rotate, changing their districts each week. The inspection of wholesale and commission houses in our city is that meat principally that has been inspected by the Federal Government known as "Interstate Meat," and the most of it comes from the large shippers and packing-houses in the West. This inspection consists in the sanitary condition of ice-boxes, wagons, cars, etc., and the cleanly condition of the meat.

I remember one time, early in my experience, my inspector reported some meat that was "off color," as he said, and upon examination I found a cargo of meat that had been killed while in an over-heated condition, and had to be condemned. The meat was absolutely rancid in twelve hours. Very few lesions of disease, if any, exist on dressed meat that could be detected by an inspector macroscopically. Therefore, the real sanitary inspection of meat comes at the slaughtering-stand; but here sometimes comes a difference of opinion. A case in point recently happened in Brooklyn in the conflict between the Federal and municipal inspectors at one of our local slaughter-houses. A cow was killed, among others, to all intents and purposes for local use, known as "kosha meat." The Government inspector was present at the slaughter and passed the cow as "all right," having localized tuberculosis in lung and glands. Our meat-inspector, who was also present, seized the carcass and condemned it for tuberculosis. The slaughter-house man refused to give it up, on the ground that it had been passed by a United States Government inspector, and the plea that he was going to send it to New Jersey, and it was, therefore, out of our jurisdiction and came under interstate inspection, and had passed that inspection. It was necessary to call in the head of the division of the Department of Health, which had charge of the meat-inspectors, to settle the matter, and it was only by forcible persuasion and a threat to revoke his permit to slaughter before the condemned carcass was given up. Had there been no United States inspector present there would have been no excuse for holding the carcass; and, on the other hand, had this happened in the absence of the local meat-inspector, as might often be the case, it would have gone through as good meat.

So, in my opinion, there should be some common rules and regulations upon which the various inspectors might stand together. It would, therefore, seem to me that a congress or convention of municipal departments of health, together with the representatives of the Federal Government, Bureau of Animal Industry, be called together for the purpose of forming some rules and regulations governing meat-inspection that would, as far as possible, decide as a unit on some definite conclusions regarding various conditions. This would have a threefold effect. In the first place, it would put all market-inspection upon the same footing; in the second place, such a convention would be a great educational feature; and, in the third place, it would have an effect upon those local boards of health who are without veterinary representatives, or it would at least draw their attention to that deficiency in their boards, and might lead to some appointments in such places.

There is one other point I want to bring up—one that I had not intended to enter into in this paper; it is a question on "tuberculosis"—and then leave the whole question of meat-inspection to this Association where it left off last year and begin with the discussion. There has been considerable dispute as to the use of meat from a tuberculous cow. It has been and still is differed about, viz.: Shall the meat of a cow with generalized tuberculosis be condemned while a cow with localized lesions be allowed to pass as good meat? The affirmative of that question has been my opinion despite the fact that our local Department of Health has instructed the meat-inspectors to take all cases of tuberculosis. The reason I ask the question now is this: I have many times seen a trimmer, by accident or design, cut into a tubercular gland of a case of localized tuberculosis, with the remark, "Oh, that don't amount to anything," and continue on the carcass with the same knife. Now, then, if we do cut down a case of localized tuberculosis it becomes a question if the butcher with his knife does not sometimes make a case of localized tuberculosis artificially a case of generalized tuberculosis by inoculating into the fascia, bones and muscular tissue the germs of tuberculosis.

Now, I sincerely trust that this question of tuberculosis, since I have mentioned it, as in most discussions on meat-inspection and milk-inspection, will not be given entirely to the disease of tuberculosis.

---

The Legislature of Georgia will be appealed to at its coming session for the granting of means and the establishing of a Livestock Sanitary Board. The livestock interests of that State are so rapidly developing that there seems an urgent need for action in this direction to properly protect the interests of those injured in this industry and for undue loss from the lack of these safeguards.

